# Changes in Leisure Self-Efficacy and Fall Risk: One-Year Results of N’Balance, a Fall Prevention Intervention Program

**DOI:** 10.1093/geroni/igaa057.953

**Published:** 2020-12-16

**Authors:** Laura Payne, Cathy Headley, Christine Katzenmeyer, Chungsup Lee

**Affiliations:** 1 University of Illinois @ Urbana-Champaign, Urbana, Illinois, United States; 2 Rockford University, Rockford, Illinois, United States; 3 Consortium for Older Adult Wellness, Lakewood, Colorado, United States; 4 California State University, Long Beach, Long Beach, California, United States

## Abstract

Fear of falling can prevent people from engaging in valued leisure activities. Yet few studies have examined the role of leisure self-efficacy in fall prevention (Datillo, Martire, Proctor, 2012). The purpose of this study was to assess how participation in a fall prevention program affected worrying about falls, self-reported falls, and leisure self-efficacy in older adults over a 1-year fall prevention intervention. N ’Balance is an 8-week community-based multi-modal fall prevention program. This community intervention study included a treatment (N=50) and control group (N=42). Data were collected in four waves: 1) pre-program physical assessment and survey, 2) post-program physical assessment and survey, 3) six month follow-up survey, and 4) 12 month follow-up survey. Measures included the Activities-Specific Balance Confidence Scale (Powell, Myers 1995), self-reported worry about falling and the leisure self-efficacy scale. Analysis of covariance was used to assess the group x time effects of N ‘Balance on worry about falling, leisure self-efficacy and the number of falls in the last 12 months, while controlling for age and subjective health. From baseline to six months post intervention, fall worry decreased significantly for the treatment group and increased for the control group (p< .05). However, there was no significant change over time in the number of self-reported falls by either group. Leisure self-efficacy was higher at baseline for the treatment group and decreased significantly from 6 to 12-months post N Balance participation, whereas the control group had lower leisure self-efficacy at baseline and increased significantly over the 1-year study period (p<.05).

